# Supplementary material for: The Natural HASPIN Inhibitor Coumestrol Suppresses Intestinal Polyp Development, Cachexia, and Hypogonadism in a Mouse Model of Familial Adenomatous Polyposis (ApcMin/+)
Source: Biology (Basel). 2024 Sep 20;13(9):736. doi: 10.3390/biology13090736 (PMC11428679; doi:10.3390/biology13090736)
Supplement: Supplementary file 1 [file biology-13-00736-s001.zip › biology-3031821-supplementary.pdf]

Table S1 Summary statistics for mice fed the standard or diet with bean sprouts.

Sex	Female				Male			
Bait type	Standard		Sprout		Standard		Sprout	
Genotype	WT	*Apc*$^{Min/+}$	WT	*Apc*$^{Min/+}$	WT	*Apc*$^{Min/+}$	WT	*Apc*$^{Min/+}$
Body weight (g)	23.1 ± 0.87	17.9 ± 1.33*	22.2 ± 1.27	20.2 ± 2.02*	28.2 ± 1.18**	18.7 ± 1.80***	25.6 ± 0.97**	24.5 ± 1.66***
Intestine length (mm)	41 ± 5.2****	38 ± 5.0	49 ± 3.3****	48 ± 3.1****	38 ± 3.4*****:	39 ± 3.2	51 ± 1.8*****	52 ± 2.7*****
No. of polyps	0	66.0 ± 11.7******	0	24.1 ± 10.7*****	0	55.8 ± 8.6*****	0	19.7 ± 13.1******

Data are means ± standard deviation ($n \geqq 6$). It was significant differences (p<0.05) between the same asterisks number (*, ** , ***, ****). ± means standard deviation. WT, wild type.

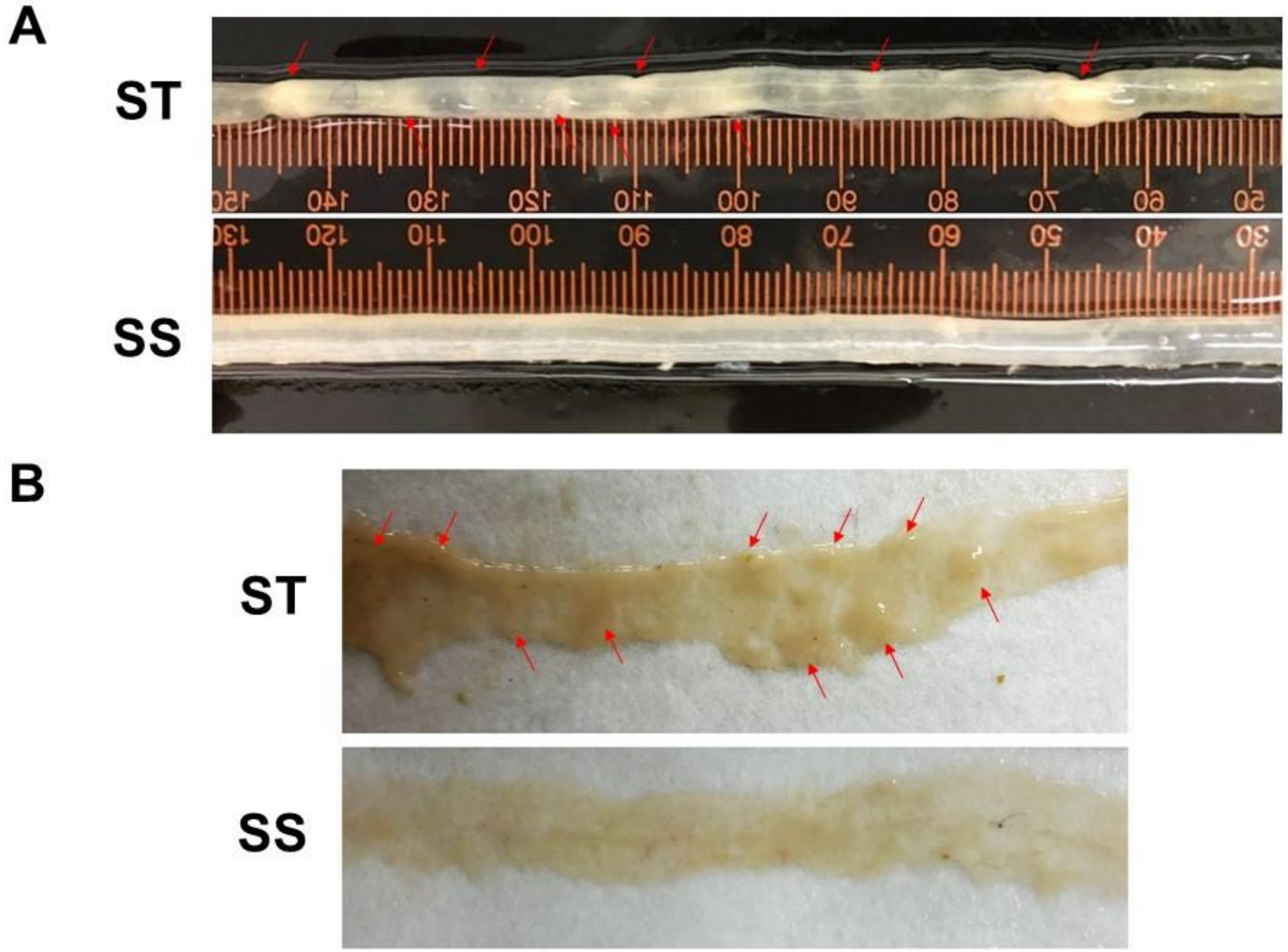

Supplementary Figure S1 Intestinal polyps of mice fed the diet with bean sprouts.

Intestinal polyps of mice fed the normal diet (ST) or a diet containing bean sprouts (SS) are shown. Polyps were not observed in the intestine containing saline (A) and cut open intestine intestne (B) of *Apc*$^{Min/+}$ mouse fed the diet with bean sprouts. Arrowes indicate polyps. Scale = 1 mm.

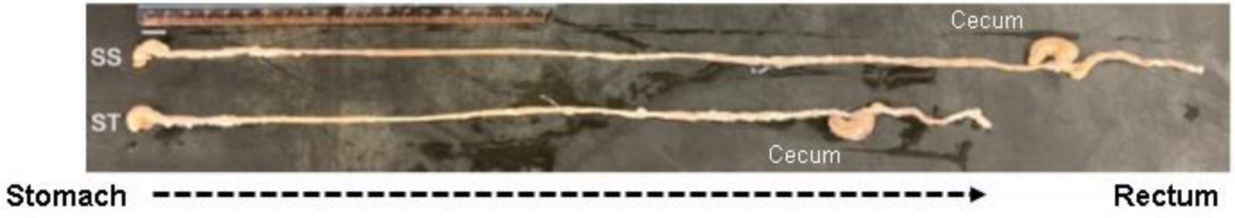

Supplementary Figure S2 Intestinal lengths of mice fed the diet with bean sprouts.

Intestines of mice fed the normal diet (ST) or a diet containing bean sprouts (SS) are shown. The intestinal length was significantly increased by the diet with bean sprouts. White bar = 10 mm.

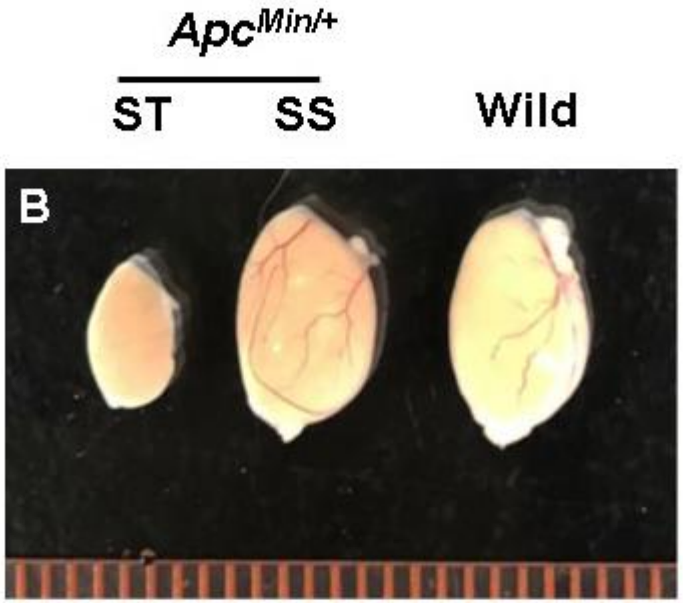

Supplementary Figure S3 Testes of mice fed the diet with bean sprouts.

Testis of *Apc*$^{Min/+}$ mice fed the normal diet (ST) or a diet containing bean sprouts (SS) are shown. Wild indicates wild type mouse fed the normal diet The intestinal length was significantly increased by the diet with bean sprouts. Scale = 1 mm.

A

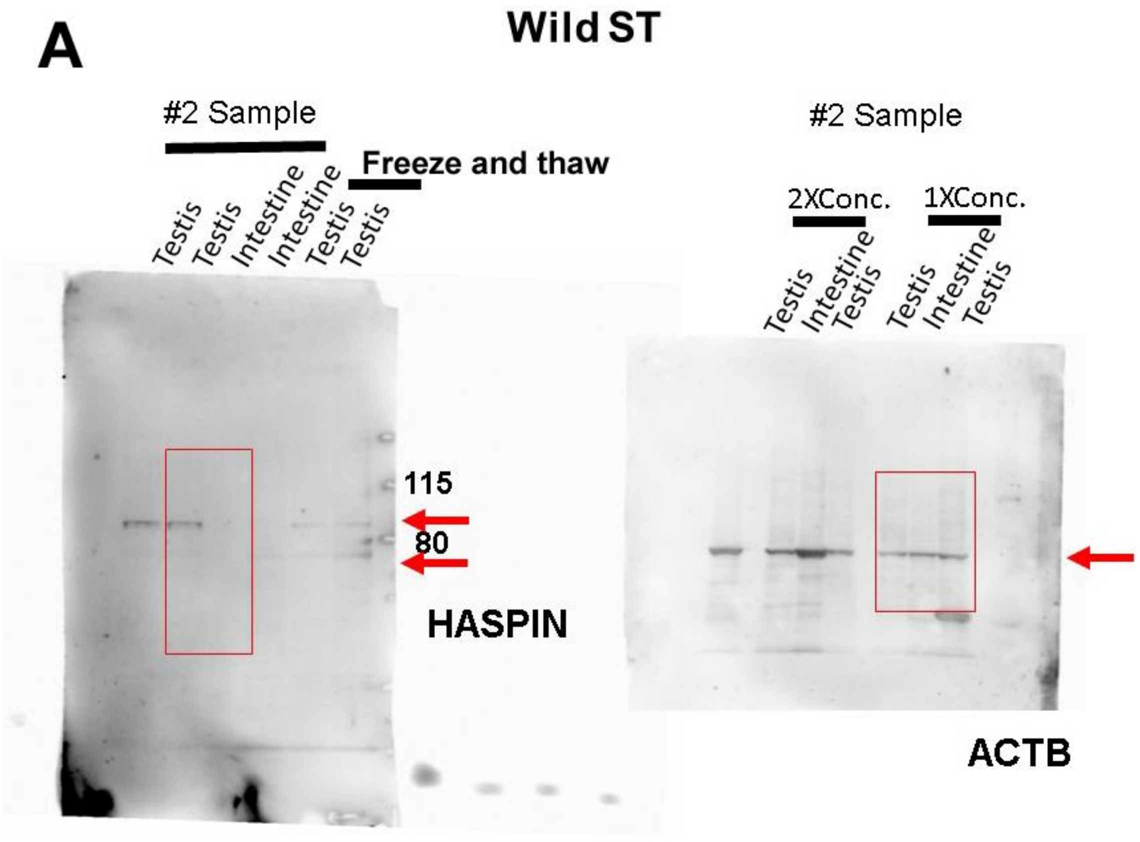
Wild ST
#2 Sample
Freeze and thaw
Testis
Testis
Intestine
Intestine
Testis
Testis
115
80
HASPIN
#2 Sample
2XConc.
1XConc.
Testis
Intestine
Testis
Testis
Intestine
Testis
ACTB

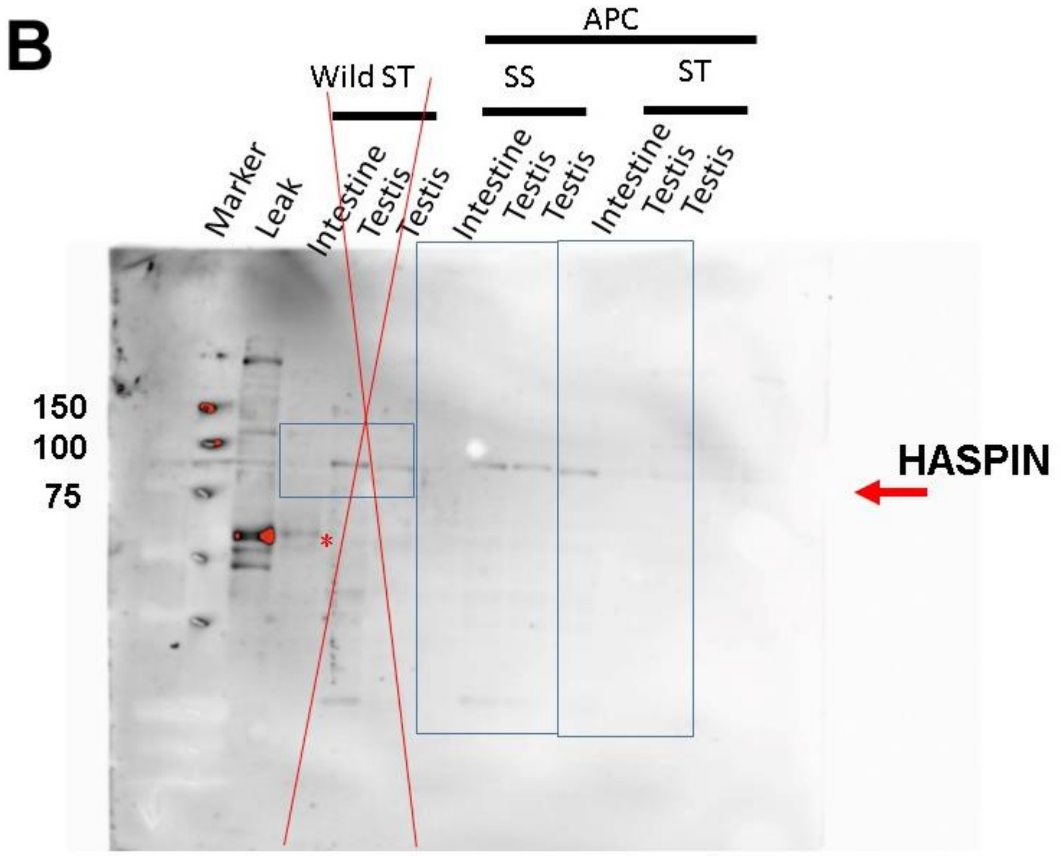
B
APC
Wild ST
SS
ST
Marker
Leak
Intestine
Testis
Testis
Intestine
Testis
Testis
Intestine
Testis
Testis
150
100
75
*
HASPIN

C

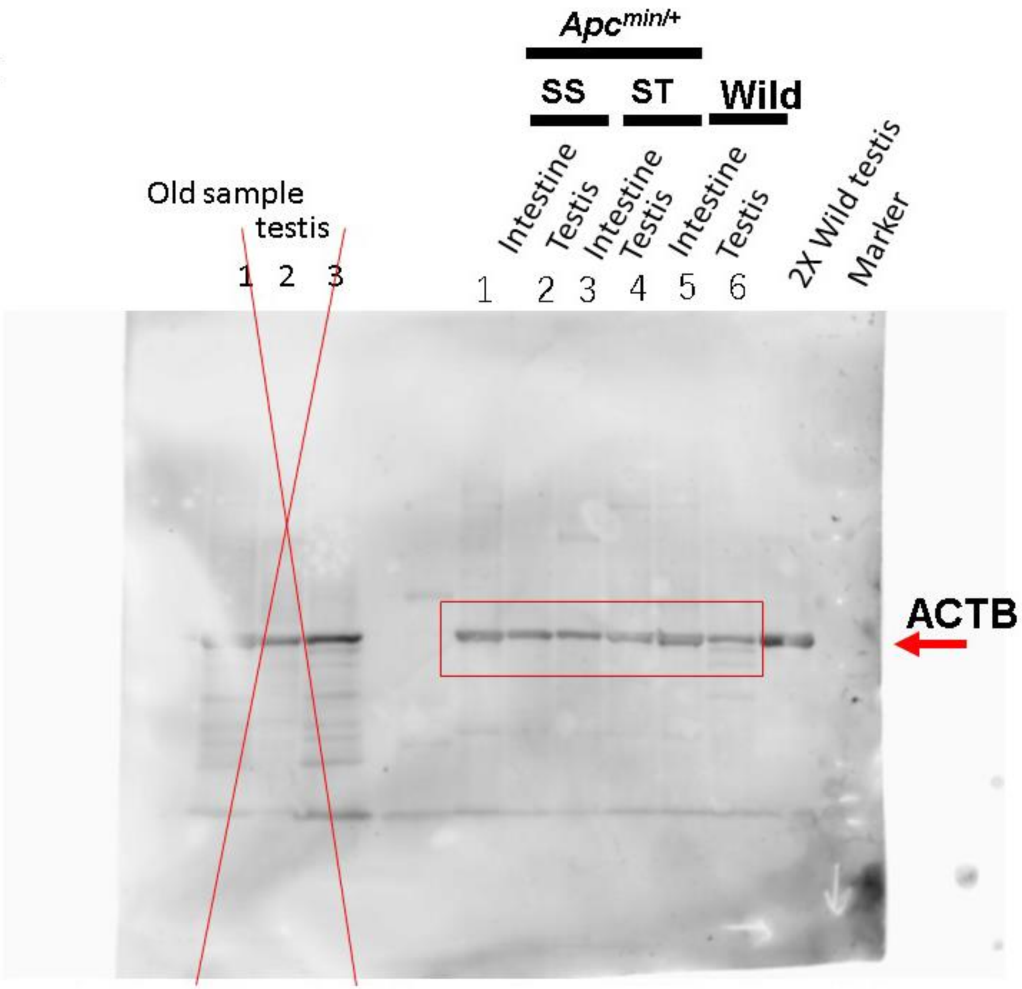

Apc^min/+
SS
ST
Wild
Intestine
Testis
Intestine
Testis
Intestine
Testis
2X Wild testis
Marker
Old sample testis
1 2 3
1 2 3 4 5 6
ACTB

Supplementary Figure S4 The uncropped western blot figures using anti-HASPIN antibody.

A. The signals of HASPIN and ACTB in the red box was used in Fig. 6. for testes and intestines of wild type mice fed the normal diet (ST). HASPIN may be easily degraded, so quantitative analysis was currently not possible with the anti-HASPIN antibody. B. The HASPIN signals in the blue box were presentated in Fig. 6. for testes and intestines of *Apc*$^{Min/+}$ mice fed the normal diet (ST) or a diet containing bean sprouts (SS). C. The ACTB signals in the red box were presentated in Fig. 6. for testes and intestines of *Apc*$^{Min/+}$ mice fed the normal diet (ST) or a diet containing bean sprouts (SS).
